# Mechanically strained osteocyte-derived exosomes contained miR-3110-5p and miR-3058-3p and promoted osteoblastic differentiation

**DOI:** 10.1186/s12938-024-01237-9

**Published:** 2024-05-05

**Authors:** Yingwen Zhu, Yanan Li, Zhen Cao, Jindong Xue, Xiaoyan Wang, Tingting Hu, Biao Han, Yong Guo

**Affiliations:** 1 Department of Biomedical Engineering, School of Intelligent Medicine and Biotechnology, Guilin Medical University, No. 1 Zhiyuan Road, Lingui District, Guilin, 541199 Guangxi People’s Republic of China; 2 Education Department of Guangxi Zhuang Autonomous Region, Key Laboratory of Biochemistry and Molecular Biology (Guilin Medical University), No. 1 Zhiyuan Road, Lingui District, Guilin, 541199 Guangxi People’s Republic of China

**Keywords:** Osteocyte, Mechanical strain, MicroRNA, Exosomes

## Abstract

**Background:**

Osteocytes are critical mechanosensory cells in bone, and mechanically stimulated osteocytes produce exosomes that can induce osteogenesis. MicroRNAs (miRNAs) are important constituents of exosomes, and some miRNAs in osteocytes regulate osteogenic differentiation; previous studies have indicated that some differentially expressed miRNAs in mechanically strained osteocytes likely influence osteoblastic differentiation. Therefore, screening and selection of miRNAs that regulate osteogenic differentiation in exosomes of mechanically stimulated osteocytes are important.

**Results:**

A mechanical tensile strain of 2500 με at 0.5 Hz 1 h per day for 3 days, elevated prostaglandin E2 (PGE2) and insulin-like growth factor-1 (IGF-1) levels and nitric oxide synthase (NOS) activity of MLO-Y4 osteocytes, and promoted osteogenic differentiation of MC3T3-E1 osteoblasts. Fourteen miRNAs differentially expressed only in MLO-Y4 osteocytes which were stimulated with mechanical tensile strain, were screened, and the miRNAs related to osteogenesis were identified. Four differentially expressed miRNAs (miR-1930-3p, miR-3110-5p, miR-3090-3p, and miR-3058-3p) were found only in mechanically strained osteocytes, and the four miRNAs, eight targeted mRNAs which were differentially expressed only in mechanically strained osteoblasts, were also identified. In addition, the mechanically strained osteocyte-derived exosomes promoted the osteoblastic differentiation of MC3T3-E1 cells in vitro*,* the exosomes were internalized by osteoblasts, and the up-regulated miR-3110-5p and miR-3058-3p in mechanically strained osteocytes, were both increased in the exosomes, which was verified via reverse transcription quantitative polymerase chain reaction (RT-qPCR).

**Conclusions:**

In osteocytes, a mechanical tensile strain of 2500 με at 0.5 Hz induced the fourteen differentially expressed miRNAs which probably were in exosomes of osteocytes and involved in osteogenesis. The mechanically strained osteocyte-derived exosomes which contained increased miR-3110-5p and miR-3058-3p (two of the 14 miRNAs), promoted osteoblastic differentiation.

**Supplementary Information:**

The online version contains supplementary material available at 10.1186/s12938-024-01237-9.

## Introduction

Mechanical loading plays an important role in regulating bone homeostasis and remodeling [1]. Moderate loading activates bone metabolism and promotes bone formation, whereas a lack of loading leads to bone resorption or disuse osteoporosis [2, 3].

Osteocytes are derived from osteoblasts, and constitute 95% of the living cells in adult bone tissue [4]. Osteocytes are critical mechanosensory cells in bone, they transform the mechanical stimulation signals into biochemical signals towards osteoblasts and osteoclasts, regulate both bone formation and resorption, and subsequently initiate bone remodeling [4–6]. After osteocytes were stimulated in vitro by mechanical loading, the conditioned medium of these cells promoted osteogenic differentiation [7–9], which indicated that some paracrine factors of the mechanically strained osteocytes, regulated osteogenic differentiation of osteoblasts or mesenchymal stem cells (MSCs).

Exosomes are formed from intracellular multivesicular bodies; they can encapsulate bioactive molecules (RNA, DNA, proteins, and so forth) and deliver the active components to adjacent or remote cells to influence the recipient cells, function [10–12]. Therefore, exosomes are mediums of intercellular communication. In recent years, osteocyte-derived exosomes had been studied. MLO-Y4 osteocytes cultured in vitro*,* could release exosomes which contain some specific miRNAs, these miRNAs were all expressed at higher level in MLO-Y4 osteocytes than in ST2 osteoblasts, and were reduced in plasma exosomes derived from osteocyte-less mice [13]. Mechanical stimulation increased exosomes production of osteocytes, and the exosomes contained sclerostin, receptor activator of nuclear factor-κ B Ligand (RANKL) and osteoprotegerin (OPG), which can influence osteogenic differentiation [14], the osteocyte exosomes contained miRNA-218 which also regulate osteogenic differentiation [15]. Moreover, the mechanically stimulated osteocytes (MLO-Y4 cells) produced exosomes which promoted osteogenesis differentiation of MSCs [16] and periodontal ligament stem cells [17].

MiRNAs are small non-coding RNA molecules which can inhibit mRNA transcription or protein translation by binding to target mRNA [18]; they play a great role in various biological activities, such as cell proliferation or differentiation, development and apoptosis, by negative regulating gene expression [19]. MiRNAs are important constituents of exosomes and strongly determine the effect of the exosomes on target cells [20, 21]. Consequently, some of the miRNAs in osteocyte-derived exosomes likely influence osteogenic differentiation. Previous studies had found that many differentially expressed miRNA in osteocytes stimulated with a mechanical tensile strain of 2500 με at 0.5 Hz, and some of the miRNAs probably regulated osteoblastic differentiation, such as miR-29b-3p [22, 23]. However, whether these miRNAs are derived from exosomes of osteocyte has not been verified. To date, the miRNAs of mechanically strained osteocyte-derived exosomes, which regulated osteoblastic differentiation, have not been screened and selected.

In this study, MLO-Y4 osteocytes and MC3T3-E1 osteoblasts were stimulated with a mechanical tensile strain of 2500 με at 0.5 Hz, respectively. After the biological responses of osteocytes to the mechanical strain and the strain-induced osteogenic differentiation of the osteoblasts were both validated, the expression profiles of miRNAs and mRNAs in the two kinds of cells were analyzed using RNA sequencing. The differentially expressed miRNAs in osteocytes (not in osteoblasts), and the targeted osteogenesis-related genes of these differentially expressed miRNAs, were screened and predicted.

MiRNAs negatively regulate their target gene mRNAs[18], high miRNA expression results in low expression of its target mRNA, and low miRNA expression leads to high expression of target mRNA. Therefore, in this study, the mechanically strained osteocyte exosome-derived miRNA were speculated through analysis of the differentially expressed miRNAs and mRNAs in the mechanically stimulated osteocytes and the stimulated osteoblasts. For example, if a miRNA is differentially expressed only in stimulated osteocytes and the miRNA target mRNA is expressed only in stimulated osteoblasts, the miRNA is likely in osteocyte-derived exosomes. The influence of mechanically strained osteocyte-derived exosomes on osteoblastic differentiation of MC3T3-E1 osteoblasts was investigated. Then, these osteocyte exosome-derived miRNAs were verified using RT-qPCR.

## Results

### Mechanical tensile strain elevated IGF-1 and PGE2 levels and increased nitric oxide synthase (NOS) activity of osteocytes

**Fig. 1:**
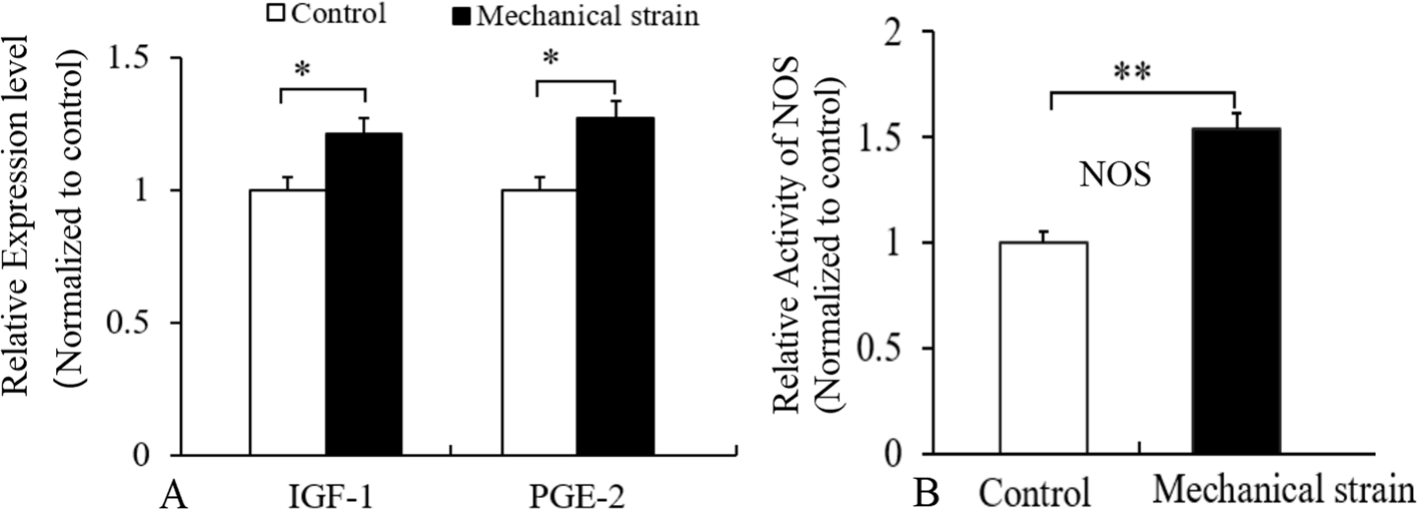
Levels of IGF-1 protein and PGE2 in the culture supernatant of MLO-Y4 osteocytes, and NOS activity in MLO-Y4 osteocytes. The ELISA result indicated that the mechanical tensile strain increased the IGF-1 and PGE2 levels of MLO-Y4 osteocytes (**A**), and increased NOS activity of the osteocytes, the NOS activity was determined via a colorimetric method (**B**). *n* = 6, *P < 0.05, ** *P* < 0.01, between the indicated groups

A mechanical tensile strain of 2500 με at 0.5 Hz for 1 h per day for 3 days increased the levels of IGF-1 protein and PGE2 (a hormone-like substance), and NOS activity in MLO-Y4 osteocytes (Fig. 1). The results indicated that the osteocytes responded to the mechanical tensile strain.

### Mechanical tensile strain promoted osteogenic differentiation of osteoblasts

**Fig. 2:**
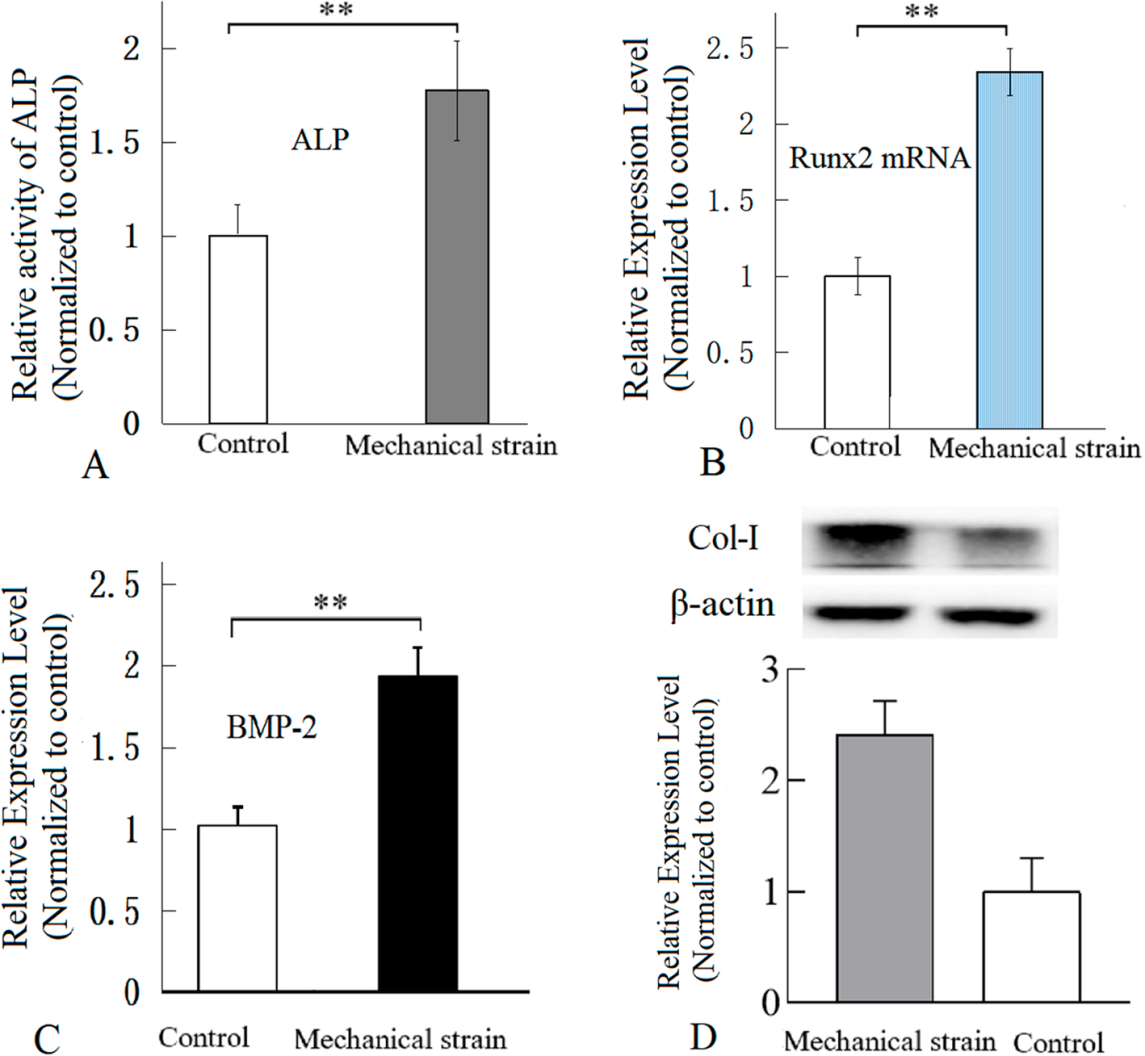
ALP activity, and the levels of BMP-2, Col-I and Runx2 in MC3T3-E1 osteoblasts. The mechanical tensile strain heightened ALP activity of MC3T3-E1 osteoblasts (**A**), up-regulated Runx2 mRNA expression in osteoblasts (as shown by RT‒qPCR) (**B**), increased BMP-2 protein level in medium of osteoblasts (as shown by ELISA) (**C**), and up-regulated protein levels of Collagen type I (as shown by Western blot) (**D**). *n* = 5, * P < 0.05, ** *P* < 0.01, between indicated groups. ALP activity was assayed with a colorimetric method

As shown in Fig. 2, the mechanical tensile strain increased the alkaline phosphatase (ALP) activity of MC3T3-E1 osteoblastic cells, increased the bone morphogenetic protein-2 (BMP-2) protein level in medium of osteoblasts, and up-regulated the protein level of collagen type I (Col-I) and mRNA level of Runx2 in osteoblasts (Additional file 1). ALP, BMP-2, Col-I and Runx2 are all the indicators of osteoblastic differentiation [24, 25], so that the results showed that the mechanical tensile strain promoted the osteogenic differentiation of osteoblasts.

### Screening of osteogenesis-related miRNAs in osteocytes

In this study, 16 miRNAs (fold change > 2 or < 0.5, *P* < 0.001) that were differentially expressed only in MLO-Y4 osteocytes which were stimulated with mechanical tensile strain (not in MC3T3-E1 osteoblastic cells) were screened out (shown in Table 1). Among the 16 miRNAs, 14 miRNAs had osteogenesis-related target genes which were predicted with TargetScan, miRanda and RNAhybrid on Majorbio Cloud Platform. Therefore, the 14 miRNAs probably were related to osteogenesis. The 14 miRNAs and their osteogenesis-related target genes are shown in Table 2.

**Table 1:** 16 miRNAs differentially expressed only in mechanically strained MLO-Y4 osteocytes (strain vs control)

miRNA_ID	Up/down	Log2(fold change)	*P* value
mmu-miR-205-5p	Up	5.10848	2.31E-20
mmu-let-7a-2-3p	Down	−1.718555	5.17E-05
mmu-miR-2137	Up	4.74591	0.002226
mmu-miR-1930-3p	Down	−3.868798	0.000244
mmu-miR-1969	Up	3.39799	1.79E-05
mmu-miR-1970b-5p	Up	1.15713	1.44E-05
mmu-miR-301b-3p	Down	−4.139445	6.49E-06
mmu-miR-3058-3p	Up	1.72283	0.000558
mmu-miR-3090-3p	Down	−2.968333	0.000661
mmu-miR-3110-5p	Up	4.63899	0.000996
mmu-miR-32-5p	Down	−4.52351	1.46E-13
mmu-miR-374b-5p	Down	−3.91798	1.03E-84
mmu-miR-467d-3p	Down	−2.935912	4.10E-05
mmu-miR-669b-5p	Down	−2.324477	0.000162
mmu-miR-7664-3p	Up	3.02602	0.000341
mmu-miR-7656-5p	Up	2.93855	0.00061

**Table 2:** Prediction of the target genes of 14 miRNAs that were differentially expressed in mechanically strained osteocytes and associated with osteogenic differentiation

MiRNA	Target gene	Gene description	Refs.
let-7a-2-3p	Arfgef1	ADP-ribosylation factor guanine nucleotide-exchange factor 1(brefeldin A-inhibited)	[26]
miR-1930-3p	Negr1	Neuronal growth regulator 1	[27]
miR-1969	Kcnmb2	Potassium large conductance calcium-activated channel, subfamily M, beta member 2	[28]
	Ranbp9	RAN binding protein 9	[29, 30]
miR-1970b-5p	Emp1	Epithelial membrane protein 1	[31]
miR-3090-3p	Adcy6	adenylate cyclase 6	[32]
miR-205-5p	Mgrn1	Mahogunin, ring finger 1	[33]
	Plcb1	Phospholipase C, beta 1	[34, 35]
miR-2137	Sgcz	Sarcoglycan zeta	[36]
	Prrx1	Paired related homeobox 1	[37, 38]
miR-374b-5p	En1	Engrailed 1	[39, 40]
	Acvr2b	Activin receptor IIB	[41]
	Pde7b	Phosphodiesterase 7B	[42]
miR-467d-3p	Cxcl5	Chemokine (C-X-C motif) ligand 5	[43, 44]
miR-669b-5p	Nfatc1	Nuclear factor of activated T cells, calcineurin dependent 1	[45, 46]
miR-7664-3p	Axin2	Axin2	[47]
miR-3110-5p	Usp34	Ubiquitin specific peptidase 34	[48, 49]
miR-3058-3p	Mapk14	Mitogen-activated protein kinase 14	[50]
miR-32-5p	Map2k4	Mitogen-activated protein kinase kinase 4	[50]
	Nox4	NADPH oxidase 4	[51, 52]

### Screening of differentially expressed miRNAs only in osteocytes and these miRNAs targeted mRNAs differentially expressed only in osteoblasts

Bioinformatics analysis on Majorbio Cloud Platform identified four differentially expressed miRNAs only in osteocytes (not in osteoblasts) (miR-1930-3p, miR-3110-5p, miR-3090-3p, and miR-3058-3p), and the four miRNAs, eight targeted mRNAs which were differentially expressed at the same time only in osteoblasts (not in osteocytes) were as follows: Apol7b, Knop1, Kctd20, Zbtb21, Mapk14, Sf1, Dgcr2, Crk (Table 3). In addition, the miRNAs that were differentially expressed only in osteoblasts and the miRNAs, targeted mRNAs that were differentially expressed only in osteocytes were not found at the same time.

**Table 3:** Differentially expressed miRNAs only in osteocytes and their targeted mRNAs which were differentially expressed only in osteoblasts at the same time

MiRNAs only differentially expressed in osteocytes	Target gene mRNA only differentially expressed in osteoblasts	Gene description
miR-1930-3p	Apol7b	Apolipoprotein L 7b
miR-3110-5p	Knop1 Zbtb21 Crk	Lysine rich nucleolar protein 1 Zinc finger and BTB domain containing 21 v-crk avian sarcoma virus CT10 oncogene homolog
miR-3090-3p	Kctd20	Potassium channel tetramerisation domain containing 20
miR-3058-3p	Mapk14 Sf1 Dgcr2	Mitogen-activated protein kinase 14 Splicing factor 1 DiGeorge syndrome critical region gene 2

### Osteocyte-derived exosomes were isolated, and absorbed by osteoblasts

**Fig. 3:**
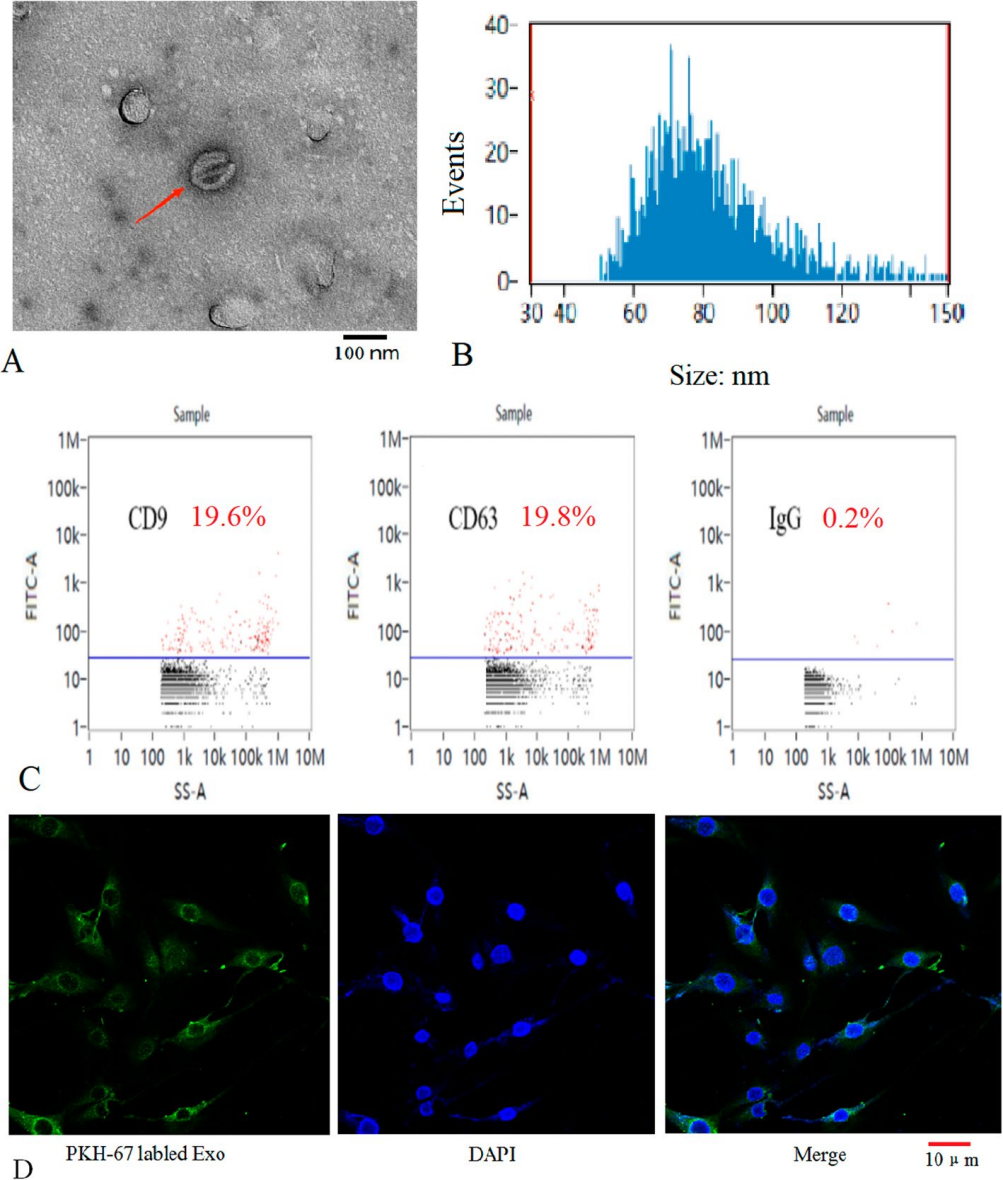
Characterization of osteocyte-derived exosomes. **A** TEM image showing the shape and size of osteocyte-derived exosomes. **B** Particle size distribution of the exosomes (as shown by NTA). **C** CD63 and CD9 were detected (the results of nanoflow analysis). **D** As shown by a laser-scanning confocal microscope, the green PKH67-labeled exosomes were absorbed by osteoblasts

Transmission electron microscopy (TEM) revealed that the osteocyte-derived extracellular particles were round shaped vesicles with membrane microcapsules, and the diameter of these vesicles was approximately 100 nm (Fig. 3A). The nanoparticle tracking (NTA) analysis result confirmed that the extracellular particles were enriched in the range of 40–160 nm, and the average diameter was approximately 80 nm (Fig. 3B). The result also showed that the particles concentration was 3.6 × 10^9^ particles/mL. In addition, using a flow NanoAnalyzer, we found that the particles released by osteocytes were positive for the exosome-specific markers CD63 (19.6% positive rate) and CD9 (19.8% positive rate) (Fig. 3C), and confocal microscopy revealed that the green PKH67-labeled exosomes were internalized by osteoblasts cultured in vitro (Fig. 3D). These results were similar to those of previous studies[53, 54]; therefore, the osteocyte-derived exosomes were isolated successfully.

### Osteoinductive potential of mechanically strained osteocyte-derived exosomes

**Fig. 4:**
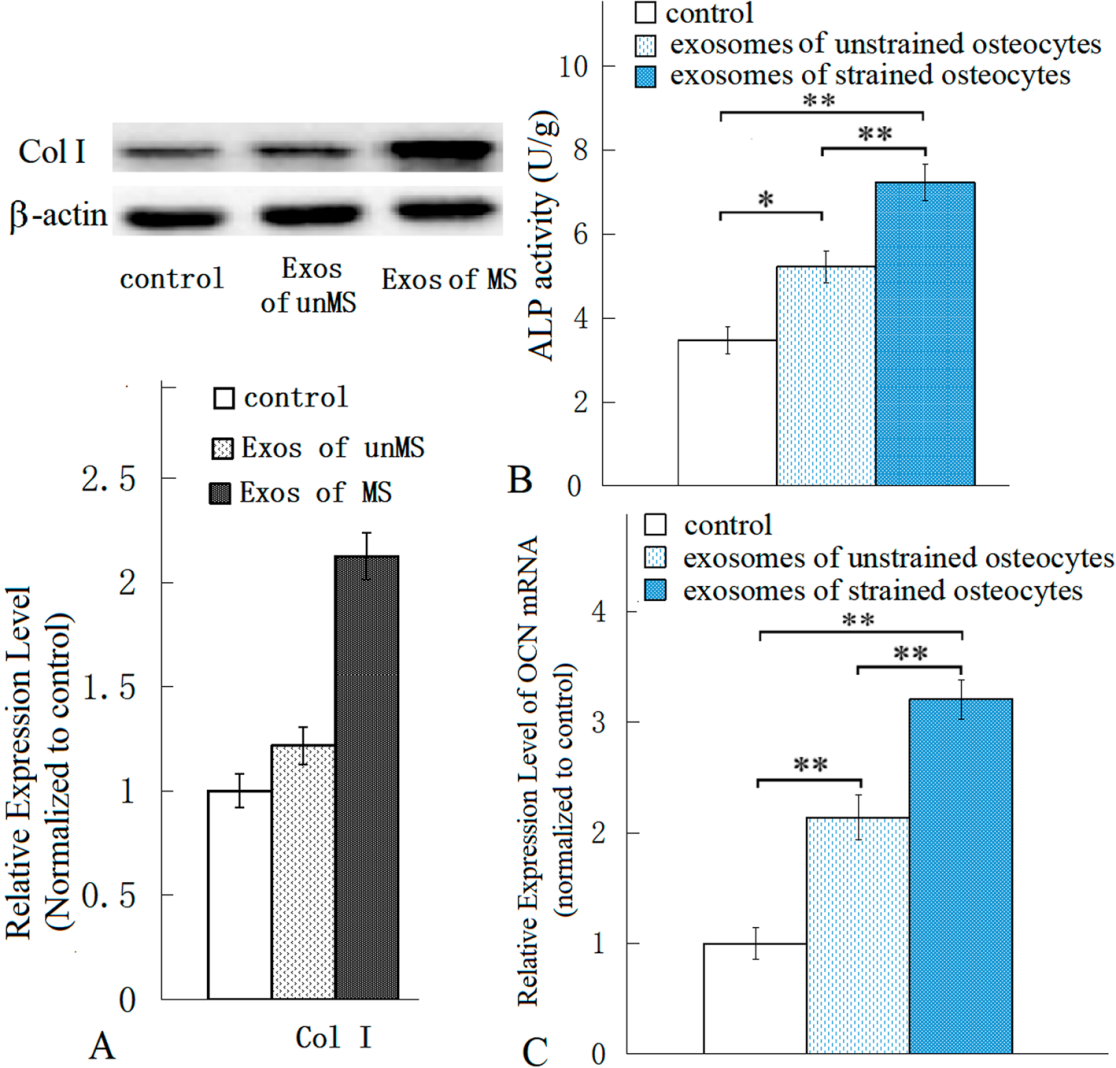
Col-I protein levels, ALP activity and OCN mRNA levels in osteoblasts treated with osteocyte-derived exosomes were assayed. After treatment with mechanically strained osteocyte-derived exosomes, the Western blot results indicated that the protein levels of Col-I in osteoblasts in vitro were increased (**A**), the ALP activity of the osteoblasts was elevated (**B**), and the RT-qPCR results showed that the OCN mRNA level in the cells was also increased (**C**). *n* = 5, * P < 0.05, ** *P* < 0.01, between the indicated groups

After treatment with mechanically strained osteocyte-derived exosomes, the protein level of Col-I in the osteoblasts cultured in vitro was increased (Fig. 4A) (Additional file 1), the ALP activity of the MC3T3-E1 cells was heightened (Fig. 4B), and the OCN mRNA in the cells was also increased (Fig. 4C). This study indicated that the mechanically strained osteocyte-derived exosomes had osteoinductive potential, and probably could promote osteoblastic differentiation of MC3T3-E1 cells in vitro*.*

### Verification of exosome-derived miRNAs

**Fig. 5:**
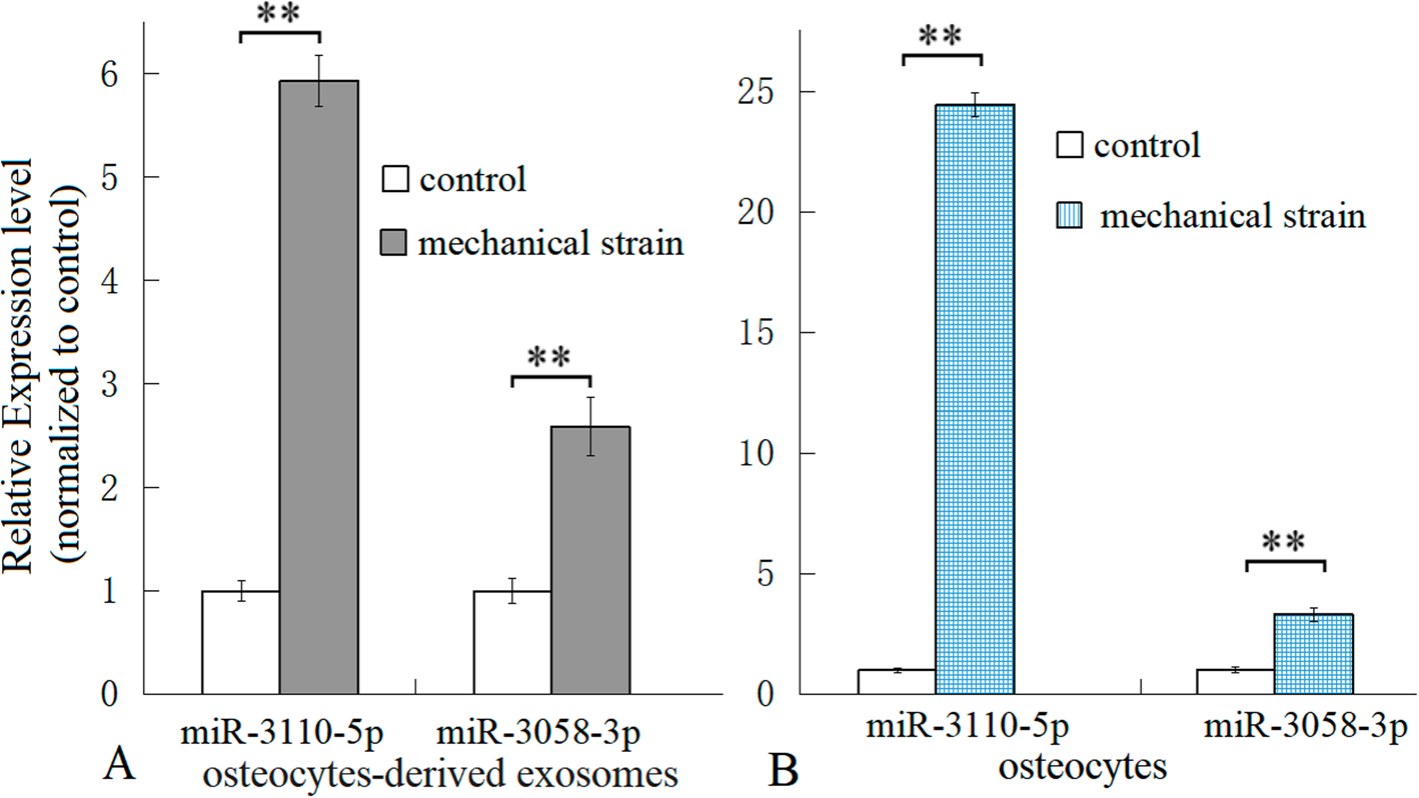
MiR-3110-5p and miR-3058-3p in osteocyte-derived exosomes and osteocytes were assayed via qPCR, respectively. The results of qPCR indicated that both in osteocyte-derived exosomes (**A**) and in osteocytes cultured in vitro (**B**), miR-3110-5p and miR-3058-3p were both up-regulated in mechanically strained osteocyte-derived exosomes and in mechanically strained osteocytes. *n* = 5, * P < 0.05, ** *P* < 0.01, between the indicated groups

The results of RNA sequencing indicated that the four miRNAs (miR-1930-3p, miR-3110-5p, miR-3090-3p, and miR-3058-3p) were differentially expressed only in mechanically strained osteocytes, and their eight targeted mRNAs were differentially expressed only in mechanically strained osteoblasts (Table 3). Both miR-3110-5p and miR-3058-3p were up-regulated, so these two miRNAs were assayed in osteocytes and osteocytes-derived exosomes with RT-qPCR. The results of RT-qPCR indicated that the mechanically strained osteocyte-derived exosomes contained more miR-3110-5p and miR-3058-3p than the unstrained osteocyte-derived exosomes (Fig. 5A). The result was consistent with the mechanical strain increasing the expressions of miR-3110-5p and miR-3058-3p in osteocytes (Fig. 5B).

## Discussion

Osteocytes are the most abundant cells in bone tissue, they detect mechanical loading, and send signals to the effector cells (osteoblast, osteoclast, and others), which mediate the formation and resorption of bone, respectively [4–6]. Osteocytes play vital roles in coordinating mechanical loading-induced bone formation via the secretion of paracrine factors [6, 55]. Exosomes are an essential route of cell–cell communication and “paracrine factor”, and miRNAs are important regulatory molecules in exosomes because of their negative regulation of gene expression [56, 57]. Therefore, in this study, the osteogenesis-related miRNAs derived from mechanically strained osteocytes, were screened and verified, and the osteoinductive potential of mechanically strained osteocyte-derived exosomes was confirmed.

In this study, after MLO-Y4 osteocytes and MC3T3-E1 osteoblasts were, respectively, stimulated with mechanical tensile strain (2500 με at 0.5 Hz, 1 h per day, for 3 days), the differentially expressed miRNAs in osteocytes (not in osteoblasts), which targeted osteogenesis-related genes, were screened, and the miRNAs derived from the exosomes of mechanically strained osteocytes were speculated via RNA sequencing and bioinformatics analysis. Then, the influence of mechanically strained osteocyte-derived exosomes on osteoblastic differentiation of MC3T3-E1 osteoblasts, was investigated, and these osteocyte exosome-derived miRNAs were verified using RT-qPCR.

The results indicated that a mechanical tensile strain of 2500 με at 0.5 Hz, promoted osteogenic differentiation of osteoblasts in vitro*,* and the mechanical tensile strain resulted in up-regulation of IGF-1, PGE2 and NOS activity, which was consistent with previous similar studies [58–61], and the IGF-1, PGE2 and NOS were involved in osteogenic differentiation [4, 6]. The results confirmed the biological response of the osteocytes and osteoblasts in vitro.

Fourteen osteogenesis-related miRNAs which were differentially expressed only in MLO-Y4 osteocytes stimulated with mechanical tensile strain, were screened, because the target genes of these miRNAs were related to osteogenesis. The 14 miRNAs were differentially expressed only in osteocytes and not in osteoblasts, so that these miRNAs probably regulated osteoblastic differentiation through the paracrine pathways, and the osteocyte-derived exosomes were the main medium.

Furthermore, in the experiment, four differentially expressed miRNAs only in osteocytes (miR-1930-3p, miR-3110-5p, miR-3090-3p, and miR-3058-3p) were selected, and the four miRNAs eight targeted mRNAs which differentially expressed only in osteoblasts were as follows: Apol7b, Knop1, Kctd20, Zbtb21, Mapk14, Sf1, Dgcr2, Crk. These eight mRNAs, which are likely regulated by the four miRNAs of osteocytes, were differentially expressed only in the mechanically strained osteoblasts, and the mechanical strain promoted osteoblastic differentiation. Therefore, the eight mRNAs probably are involved in mechanical stimulation induced osteogenesis and the four miRNAs of osteocytes, are likely to influence osteoblastic differentiation through exosomes.

In response to mechanical stimulation, such as fluid flow stress, osteocytes released signaling molecules and paracrine factors, such as PGE2, NO, IGF-1, and OPG, to promote osteogenesis [4, 6, 22]. Exosomes play a key role in the paracrine pathway, they contain numerous biological molecules including miRNAs, and directly participate in signal communication between cells [56, 57]. Some studies had shown that osteocyte-derived exosomes promoted the osteoblastic differentiation or bone formation, some miRNAs in the exosomes, such as miR-218 and miR-124-3p, were likely involved in osteogenesis [18, 61]. Interestingly, the exosomes derived from MLO-Y4 cells subjected to mechanical cyclic stretch of 8%, induced osteogenic differentiation of human periodontal ligament mesenchymal stem cells, and the exosomal miR-181b-5p of the osteocytes, was involved in osteogenic differentiation [17]. Exosomes isolated from osteocytes which were activated with fluid shear stress, also enhanced the osteogenic differentiation of hMSCs [16].

The results of the study indicated that the exosomes of the mechanically strained osteocytes could induce osteogenic differentiation of osteoblasts, the up-regulated miR-3110-5p and miR-3058-3p were confirmed to be present in the exosomes of mechanically stimulated osteocytes, and the two exosomal miRNAs probably influenced osteogenic differentiation. Fourteen osteogenesis-related miRNAs (including miR-3110-5p and miR-3058-3p) which were differentially expressed only in osteocytes subjected to the mechanical strain, that were likely present in the exosomes of osteocytes, and regulated osteogenic differentiation of osteoblasts via exosomes.

Through bioinformatic analysis of the differentially expressed miRNAs and mRNAs of osteocytes and osteoblasts which were both stimulated with mechanical strain, the exosomal osteogenesis-related miRNAs of osteocytes were selected. This study provided a novel means to predict exosomal osteogenesis-related miRNAs in osteocytes. Two miRNAs (miR-3110-5p and miR-3058-3p) in the exosomes of mechanically strained osteocytes were confirmed via RT-qPCR, and the results of the study indicated that the exosomes were internalized by osteoblasts, and the exosomes induced osteogenic differentiation. Therefore, in this study, the exosomes of the mechanically stimulated osteocytes, probably delivered these 14 osteogenesis-related miRNAs (at least miR-3110-5p and miR-3058-3p) to osteoblasts and regulated the osteogenic differentiation of the cells.

## Conclusion

In osteocytes, a mechanical tensile strain of 2500 με at 0.5 Hz induced differential expression of the 14 miRNAs which were likely present in the exosomes of osteocytes and were involved in osteogenesis. The exosomes of osteocytes which were stimulated with the mechanical tensile strain, promoted the osteogenic differentiation of osteoblasts and delivered miR-3110-5p and miR-3058-3p (two of the 14 miRNAs) to osteoblasts.

## Materials and methods

### Cell culture and application of mechanical strain

Mouse MLO-Y4 osteocyte-like cells and MC3T3-E1 osteoblastic cells (Guangzhou JENNIO Biological Technology, China) were cultured, respectively, in dishes with a-MEM medium (α-MEM, Invitrogen) supplemented with 10% FBS and 1% penicillin–streptomycin (Invitrogen). Then, the MLO-Y4 cells and MC3T3-E1 cells were, respectively, seeded into polystyrene loading dishes of a stepping motor-derived four-point bending device which could produce homogenous, tensile strains to the substrate of the mechanical loading dishes [62–64]. When these cells were at confluence, the culture medium supplemented with 10% exosome-free FBS was renewed, the MLO-Y4 cells and MC3T3-E1 cells in the loading dishes (or cell carrier) [64] were stimulated separately with a mechanical tensile strain of 2500 με (at 0.5 Hz, 1 h per day, for 3 days), which was generated by the four-point bending device. Previous studies indicated that the mechanical tensile strain could promote osteoblast differentiation [65, 66].

### Enzyme-linked immunosorbent assay (ELISA)

After mechanical stimulation, the levels of PGE2 hormone and IGF-1 protein in the culture supernatant of MLO-Y4 cells were assayed separately using a PGE2 ELISA kit and an IGF-1 ELISA kit (Elabscience Biotechnology Co., Ltd., Wuhan China) according to the manufacturers, instructions.

### Assay of NOS activity

After mechanical stimulation, the MLO-Y4 cells were treated with 0.1% triton X-100 for 30 min, then the NOS enzyme activity in the cells lysates was measured with a colorimetric method using a NOS detection kit (Nanjing Jiancheng Bioengineering Institute, China) according to the protocol of the supplier.

### Assay of ALP activity

The MC3T3-E1 cells were lysed by sonication at 25 kHz for 1 min on ice in radioimmunoprecipitation (RIPA) lysis buffer (Beyotime Biotechnology, ShangHai, China). The protein concentration of the cell lysates was assayed using a bicinchoninic acid protein assay kit (Beyotime Biotechnogy). Then the ALP enzyme activity in the cell lysates was assayed using an alkaline phosphatase assay kit (Beyotime Biotechonogy), according to the supplier's protocol (http://www.beyotime.com/product/P0321S.htm).

### Western blot

After mechanical tensile strain, the cells were lysed in RIPA buffer solution (Beyotime), and the protein in the cell lysates was quantified using the BCA method. The protein in the lysates was separated by electrophoresis in 12% polyacrylamide gel containing 0.15% sodium dodecyl sulfate, then transferred onto PVDF membranes (Millipore, USA). After blocking with 5% skim milk-TBST and incubation with primary antibodies overnight at 4 °C, the membranes were incubated with horseradish peroxidase conjugated secondary antibody. The reactive bands were visualized using an enhanced chemiluminescent substrate solution (Beyotime Biotechnology) and analyzed using the ImageJ software (http://imagej.nih.gov/ij/).

### Sequencing of mRNA and miRNA

After total RNA of the cells was isolated with TRIzol reagent (Invitrogen), the purity of the RNA was assessed using the ND-2000 Nanodrop (Thermo Fisher Scientific), and the integrity of RNA was evaluated using the 2100 Bioanalyzer (Agilent Technologies, Inc.). Then, the RNA sequencing and subsequent bioinformatics analysis were performed and provide by Shanghai Majorbio Bio-Pharm Technology Co., Ltd. (https://www.majorbio.com/majorbio/index). The differential expression analysis of mRNAs in cells was conducted using the DEGseq R package (http://bioinfo.au.tsinghua.edu.cn/software/degseq), and the differential expression analysis of miRNA in the cells was performed using miRDeep2 (v2.0.0) software [67, 68].

### Screening of osteocyte-derived differentially expressed miRNAs and these miRNAs, target mRNAs differentially expressed in osteoblasts

After the differentially expressed miRNAs and mRNAs of the mechanically strained osteocytes and MC3T3-E1 osteoblasts were obtained (fold change > 2 or < 0.5, *P* < 0.01), the differentially expressed osteocyte-derived miRNAs targeted the differentially expressed mRNAs of the MC3T3-E1 osteoblasts, which were predicted at the same time with TargetScan, RNAhybrid and miRanda, were identified. These differentially expressed miRNAs only in osteocytes and these miRNAs, target mRNAs differentially expressed only in osteoblasts, were screened and identified. The differentially expressed osteoblast-derived miRNAs targeted differentially expressed mRNAs of osteocytes, which were also predicted with the same method, were also screened. Then the differentially expressed miRNAs only in osteoblasts and these miRNAs, target mRNAs differentially expressed only in osteocytes, were also selected. All of these bioinformatics analyses and predictions were performed online using the Majorbio Cloud Platform (www.majorbio.com).

### RT-qPCR for mRNA and miRNA

After total RNA of the cells was extracted with TRIzol reagent, cDNA was synthesized using a Quant RT kit (Tiangen Biochemistry Co., Ltd, Beijing, China), according to the manufacturer's protocol. The mRNA expression levels were determined using SYBR Green qPCR Premix (Tiangen Biochemistry) on an Applied Biosystems, Real-Time PCR system (7500, Thermo Fisher Scientific Inc), according to the manufacturer's instructions. The reaction process included denaturation at 95 °C for 3 min, followed by 40 cycles of 95˚C for 15 s, 60 °C for 30 s. The mRNA levels were normalized to those of the internal control β-actin.

The miRNA expression levels were assessed using the All-in-One™ miRNA RT-qPCR Detection Kit 2.0 (GeneCopoeia, Inc. Guangzhou, China) according to manufacturer,s protocol, the primers for RT-qPCR were synthesized by manufacturer according to the miRNA sequences listed in the Sanger miRBase, with U6 serving as the reference gene (internal control). The reactions were incubated in a 96-well optical plate at 95 °C for 9 min, followed by 40 cycles of 15 s at 95 °C, 20 s at 60 °C and 20 s at 72 °C C, then 72 °C for 4 min.

### Detection of osteoblastic differentiation

To determine osteoblastic differentiation of MC3T3-E1 cells, the ALP activity of the cells was assayed with the ALP kit mentioned above, the protein level of Collagen type I (Col-I) was detected via Western blot, and the mRNA levels of osteocalcin (OCN) and Runx2 were assayed via RT-qPCR, The methods of Western blot and RT-qPCR were described above, the primers of OCN were as follows, forward:

AGTCTGACAAAGCCTTCA, reverse:AAGCAGGGTTAAGCTCACA; the primers of Runx2 were as follows, forward: AGTAGCCAGGTTCAACGAT, reverse: GGAGGATTTGTGAAGACTGTT. In addition, the Bone morphogenetic protein-2 (BMP-2) protein level in the culture supernatant of the cells was detected via ELISA also mentioned above.

### Isolation and identification of exosomes from MLO-Y4 osteocytes

The isolation and identification of the exosomes were performed as previously described [53, 54, 69]. After the osteocytes were cultured in exosome-free medium for 48 h on mechanical loading dishes, the culture medium was collected and subjected to 300 × g centrifugation for 10 min and 2000×*g* centrifugation for 15 min. After that, the supernatant was obtained by filtration using a 0.45-μm filter to eliminate cell debris, and was ultracentrifuged twice at a speed of 100,000×*g*, at 4 °C for 70 min (after the first ultracentrifugation, each tube was washed with 5 ml PBS and then filtered through a 0.22-μm membrane filter). The final pellet (pure exosomes) was resuspended in 200 μl PBS and stored at −80 °C.

The diameter of the exosomes was measured by NTA using a ZetaView Particle Metrix (Particle Metrix, Meerbusch, Germany). The shape and size of the exosomes were observed by TEM (FEI Tecnai G2 Spirit BioTwin; FEI, Hillsboro, OR, USA). Exosome-positive markers (CD9, CD63) were detected by Flow NanoAnalyzer (N30E, NanoFCM INC, Xiamen, China).

### Exosomes uptake assay

The uptake assay was performed as previous described [69, 70], the exosomes were labeled with 10 μM PKH67 (a green fluorescent dye, Sigma Aldrich), and incubated in the dark at room temperature for 12 min, and the excess dye was removed using a 100kD ultrafiltration device (Millipore, USA), then the exosomes resuspended in culture medium. Subsequently, the PKH67-labelled exosomes were added to MC3T3-E1 cells and incubated for 24 h at 37◦C. After the cells were fixed with 4% formaldehyde, the nuclei of the cells were stained with 4', 6-diamidino-2-phenylindole (DAPI, Sigma Aldrich), and the cells were visualized by fluorescence under a laser-scanning confocal microscope (LSM710 Carl Zeiss AG).

### Statistical analysis

All the data were showed as the mean ± standard deviation. Normal distribution of data was tested using the Shapiro–Wilk test, and differences between groups were analyzed using one-way analysis of variance. Statistical analysis was performed using SPSS software (version 19; SPSS, Inc.) and *P* < 0.05 was considered to indicate a statistically significant difference.

### Supplementary Information

**Additional file 1.** Uncropped western blot image of Figure 2D

**Additional file 2.** Uncropped western blot image of Figure 4A

## Data Availability

The dataset(s) supporting the conclusions of this article is(are) included within the article.

## Abbreviations

miRNA: MicroRNA
PGE2: Prostaglandin E2
IGF-1: Insulin-like growth factor-1
NOS: Nitric oxide synthase
RT-qPCR: Reverse transcription quantitative polymerase chain reaction
MSCs: Mesenchymal stem cells
RANKL: Receptor activator of nuclear factor-κB ligand
OPG: Osteoprotegerin
ELISA: Enzyme-linked immunosorbent assay
ALP: Alkaline phosphatase
RIPA: Radioimmunoprecipitation
BCA: Bicinchoninic acid
Runx2: Runt-related transcription factor 2
Col-I: Collagen type I
OCN: Osteocalcin
BMP-2: Bone morphogenetic protein-2
NTA: Nanoparticle tracking analysis
