# Supplementary material for: Mechanically strained osteocyte-derived exosomes contained miR-3110-5p and miR-3058-3p and promoted osteoblastic differentiation
Source: Biomed Eng Online. 2024 May 5;23:44. doi: 10.1186/s12938-024-01237-9 (PMC11070085; doi:10.1186/s12938-024-01237-9)
Supplement: Supplementary file 1 — Additional file 1. Uncropped western blot image of Figure 2D [file 12938_2024_1237_MOESM1_ESM.docx]

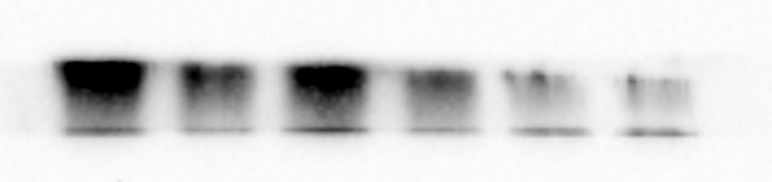


Col-I


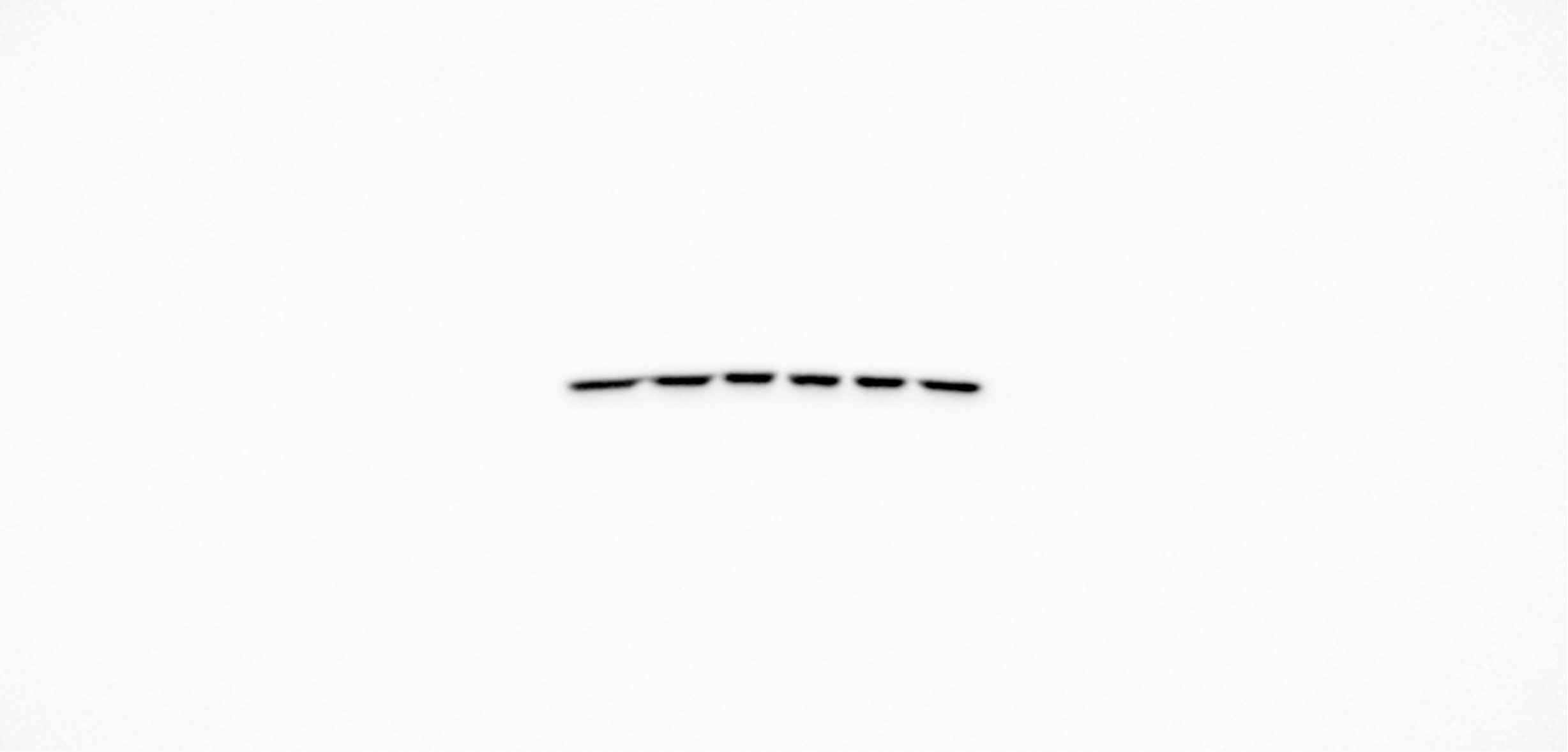


β-actin

Strain strain strain control control control

Uncropped western blot image of Figure 2D
