# Supplementary material for: Mechanically strained osteocyte-derived exosomes contained miR-3110-5p and miR-3058-3p and promoted osteoblastic differentiation
Source: Biomed Eng Online. 2024 May 5;23:44. doi: 10.1186/s12938-024-01237-9 (PMC11070085; doi:10.1186/s12938-024-01237-9)
Supplement: Supplementary file 2 — Additional file 2. Uncropped western blot image of Figure 4A [file 12938_2024_1237_MOESM2_ESM.docx]

β-actin Col-I


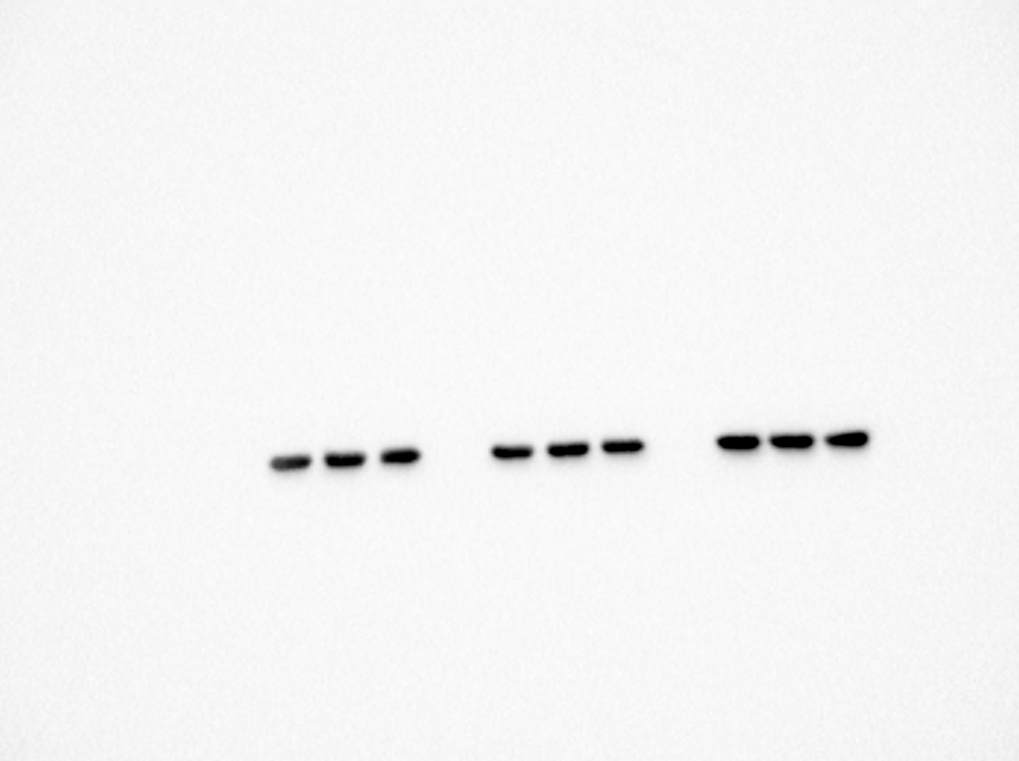

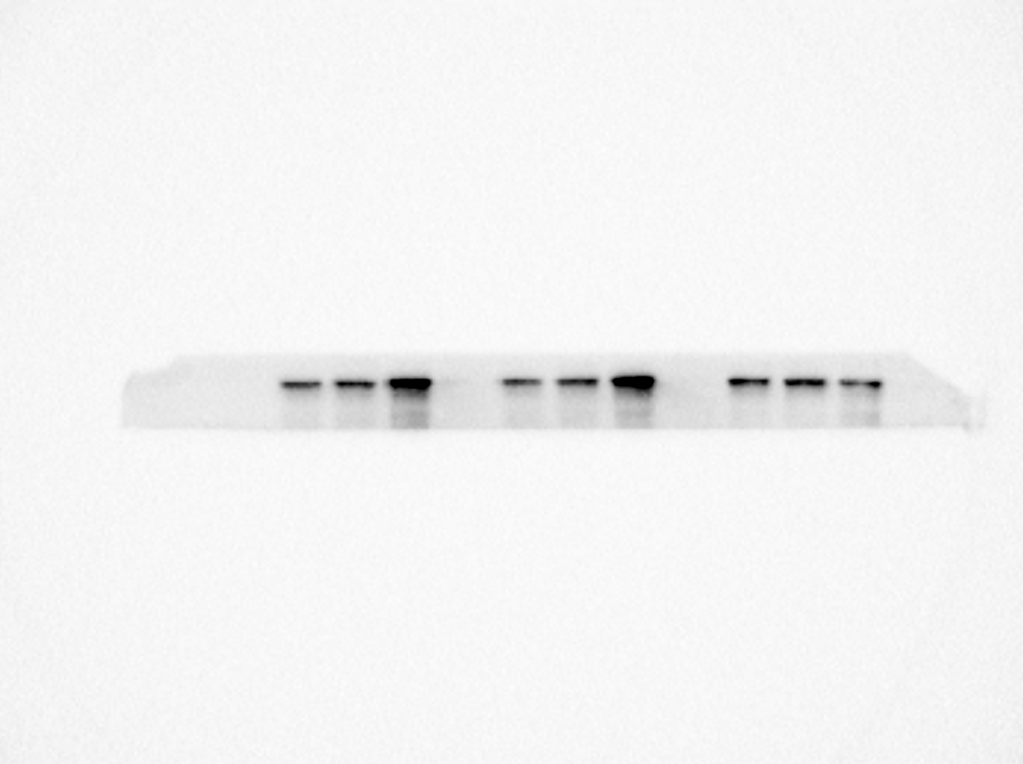


From left to right of each image (control, Exos of unstrain, Exos of strain), (control, Exos of unstrain, Exos of strain), ( Exos of unstrain, Exos of strain,control ),

Uncropped western blot image of Figure 4A
